# Non‐parallel anti‐tumour effects of pembrolizumab: a case of cardial tamponade

**DOI:** 10.1002/rcr2.404

**Published:** 2019-01-31

**Authors:** Motoko Tachihara, Masatsugu Yamamoto, Masako Yumura, Asuka Yoshizaki, Kazuyuki Kobayashi, Yoshihiro Nishimura

**Affiliations:** ^1^ Division of Respiratory Medicine, Department of Internal Medicine Kobe University Graduate School of Medicine Kobe Japan

**Keywords:** Lung adenocarcinoma, pembrolizumab, pericardial effusion, cardial tamponade

## Abstract

We present the case of a 70‐year‐old man with stage IV lung adenocarcinoma. He was treated with pembrolizumab, a programmed cell death‐1 (PD‐1) inhibitor, as a first‐line therapy. After six cycles of pembrolizumab, he suddenly developed cardiac tamponade. With the exception of newly massive malignant pericardial effusion, the other malignant lesions improved. Pembrolizumab was continued and the patient has shown a durable response for two years. This is the unique case of late‐onset pericaridial effusion with pembrolizumab, showed discrepant anti‐tumour effects. A proper assessment is crucial to ensure favourable clinical outcomes in patients treated with PD‐1 inhibitor.

## Introduction

Pembrolizumab, an anti‐programmed cell death‐1 (PD‐1) protein monoclonal antibody, has anti‐tumour activity in advanced non‐small‐cell lung cancer. In patients with advanced non‐small cell lung carcinoma (NSCLC) in which at least 50% of the tumour cells express PD‐L1, pembrolizumab is associated with significantly longer progression‐free and overall survival in comparison to chemotherapy. In clinical practice, the use of pembrolizumab has rapidly increased. On the other hand, immune checkpoint inhibitors (ICIs) such as pembrolizumab can induce specific immune‐related adverse events (irAEs). When pericardial fluid appears or increases, physicians may be unsure whether it represents an exacerbation of the disease or an irAE. In addition, physicians may be unsure of whether to continue treatment when a newly developed malignant fluid appears in the body cavity when the other lesions have improved.

We describe the unique case of non‐parallel anti‐tumour phenomena of pembrolizumab in a patient with lung adenocarcinoma who developed disease progression with cardiac tamponade alone. The patient was able to continue treatment with pembrolizumab after the only drainage of massive volume of malignant effusion and he showed a durable response to pembrolizumab.

## Case Report

**Figure 1:**
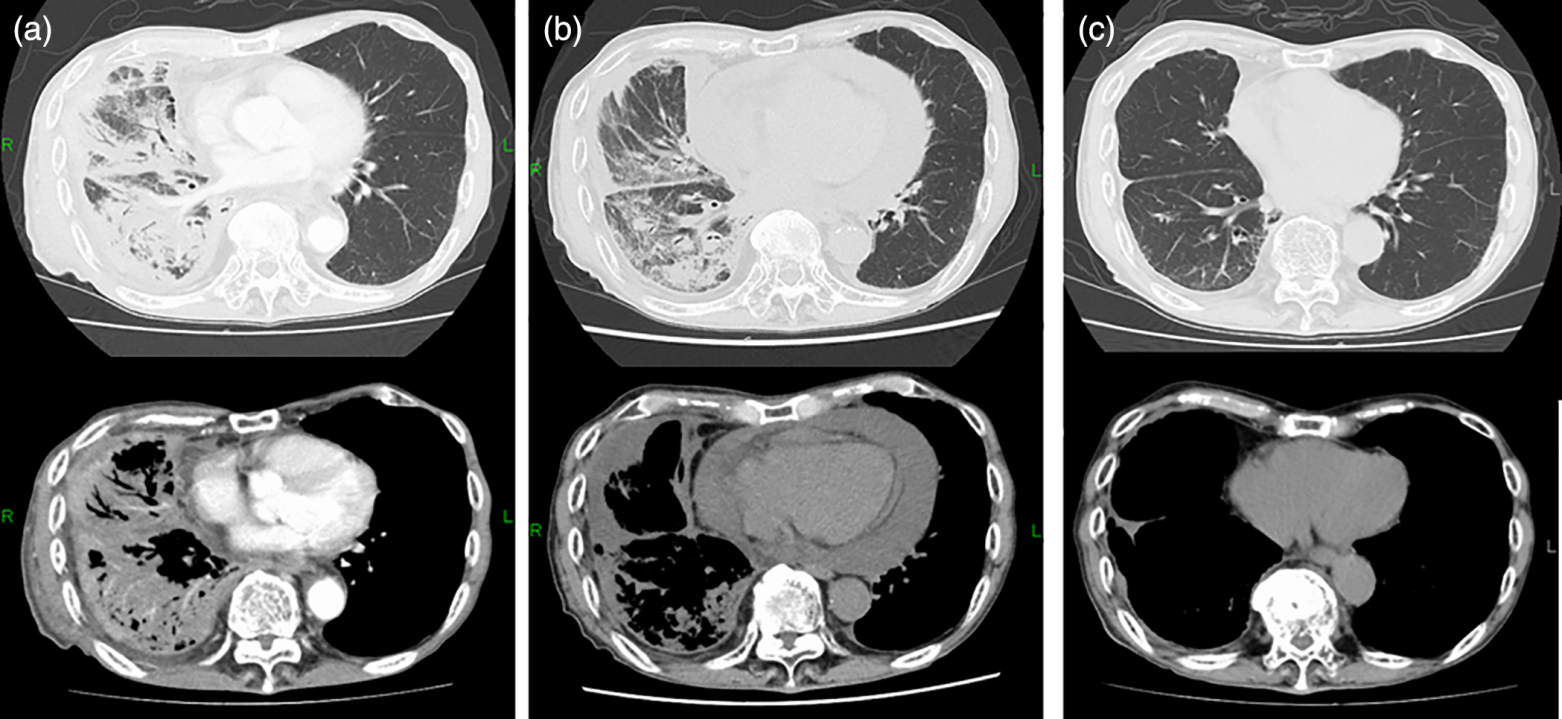
Chest computed tomography (CT). (A) Prior to treatment with pembrolizumab. CT showed carcinomatous lymphangitis and pleural effusion. The primary site was not clear under cover to lymphangitis. (B) After six cycles. A massive pericardial effusion was observed. The other malignant lesions improved. (C) After 11 cycles. The patient showed a continuous good response.

A 70‐year‐old man patient with a 40‐year smoking history presented with dyspnoea. Chest X‐ray and a computed tomography (CT) scan showed massive pleural effusion. He was subsequently diagnosed with stage IV lung adenocarcinoma (cT3N3M1b) according to the analysis of the pleural effusion. The tumour was negative for epidermal growth factor receptor mutations and anaplastic lymphoma kinase gene rearrangement. More than 90% of the tumour cells expressed PD‐L1. He had an Eastern Cooperative Oncology Group (ECOG) performance‐status score of 2. First‐line treatment with pembrolizumab was initiated at the standard dosage (200 mg/body, tri‐weekly), following the drainage of the pleural effusion. After two cycles and four cycles of treatment with pembrolizumab, a CT scan showed a good response in the primary lesion and carcinomatous lymphangitis of the right lung, and the volume of pleural effusion was decreased. Pericardial effusion was not observed from the time of diagnosis. After six cycles of treatment with pembrolizumab, he was suddenly admitted to our emergency clinic with dyspnoea and general fatigue. A physical examination revealed the following: blood pressure, 112/85 mmHg; heart rate, 114 beats/min, respiratory rate, 20 breaths/min; O_2_ saturation, 97% (with 2 L/min of O_2_ by nasal cannula); and temperature, 36.4 °C. Chest X‐ray showed cardiomegaly. Chest CT showed a newly developed massive pericardial effusion; however, the anti‐tumour effect in the primary tumour and lymphangitis were maintained (Fig. 1A, B). Electrocardiography showed a low QRS voltage and complete right bundle branch block with left axis deviation. Echocardiography showed a large echo‐free space around the heart and the collapse of the right atrium and ventricle, consistent with pericardial tamponade. Subsequent aggressive fluid resuscitation was initiated. Pericardiocentesis was performed and 480 mL of blackish‐brown fluid was drained; a drainage tube was then placed. An analysis of the pericardial fluid revealed the following: lactate dehydrogenase, 269 IU/L; protein, 5.3 g/dL; glucose, 75 mg/dL; carbohydrate antigen (CA) 19‐9, 2245 U/mL; pH, 7.301; and total cell count, 4625/μL with 26% mononucleocytes and 27% polymorphonucleocytes. A cytological examination of the pericardial fluid showed adenocarcinoma. Gram staining and bacterial culturing revealed no microorganisms. Chest X‐ray showed the resolution of the pericardial effusion and the drainage tube was removed two days later. The patient showed progressive disease with newly emergent pericardial malignant effusion; however, the other malignant lesions improved. Finally, we decided to continue treatment with pembrolizumab. The patient showed a continuous response after 18 cycles of treatment without any further pericardial effusion (Fig. 1C).

## Discussion

ICIs such as pembrolizumab and nivolumab represent a paradigm shift for the therapy for NSCLC. ICIs show a durable clinical benefit, but they are also associated with toxicities, which are known as irAEs. Serious and life‐threatening irAEs have been reported, including pneumonitis and hypophysitis. There have been five reports on pericardial effusion induced by ICIs (ipilimumab, *n* = 2; nivolumab, *n* = 3) 1, 2, 3, 4, 5. Most of these reports are pericardial effusion being induced by irAEs 1, 2, 3, 4. In these cases, pericardial fluid cytology showed a lymphocytic predominance, with no evidence of malignancy. Pericardial tissue biopsy revealed acute inflammation, suggesting that ICIs induced acute immune‐related pericarditis and pericardial effusion 1, 2, 4. Most of these cases occurred within three months after the initiation of therapy and subsequent steroid therapy was reported to be effective. Kolla and Patel reported two cases of recurrent pleural effusion and cardiac tamponade in association with nivolumab 5. These patients originally had a history of carcinomatous pericarditis and the development of cardiac tamponade was consistent with the possible manifestations of pseudoprogression. In our case, the patient did not have any malignant pericardial effusion at the time of the diagnosis, and it newly appeared later in the course of treatment with pembrolizumab. There was an apparent discrepancy between the rapid appearance of pericardial malignant fluid and the other lesions. In this case, it is critical to determine whether ICI treatment should be continued or discontinued. If newly developed malignant effusion occurs in the body cavity, we judge it as progressive disease and treatment is usually terminated. In ICI‐treated patients, however, it may be possible to continue ICI treatment if other lesions show improvement. I guess why this non‐parallel anti‐tumour effect phenomena occurred was caused by different drug response by the intratumour heterogeneity due to the difference in the tumour microenvironment or drug transferability to tissues.

In conclusion, we described the unique case of late‐onset pericardial tamponade with pembrolizumab as a non‐parallel anti‐tumour effect. There is a need to discuss the appropriate assessment and the administration of ICIs by collecting more cases as this may affect the prognosis of patients with NSCLC.

### Disclosure Statement

Appropriate written informed consent was obtained for publication of this case report and accompanying images.
